# Development and Testing of a Computerized Decision Support System to Facilitate Brief Tobacco Cessation Treatment in the Pediatric Emergency Department: Proposal and Protocol

**DOI:** 10.2196/resprot.4453

**Published:** 2016-04-20

**Authors:** E. Melinda Mahabee-Gittens, Judith W Dexheimer, Jane C Khoury, Julie A Miller, Judith S Gordon

**Affiliations:** ^1^ Cincinnati Children's Hospital Medical Center Division of Emergency Medicine Cincinnati, OH United States; ^2^ Cincinnati Children's Hospital Medical Center Division of Emergency Medicine and Department of Biomedical Informatics Cincinnati, OH United States; ^3^ Cincinnati Children's Hospital Medical Center Division of Biostatistics and Epidemiology Cincinnati, OH United States; ^4^ University of Arizona Department of Family & Community Medicine Tucson, AZ United States

**Keywords:** smoking, tobacco smoke, parent, secondhand smoke, medical informatics, clinical decision support, smoking cessation

## Abstract

**Background:**

Tobacco smoke exposure (TSE) is unequivocally harmful to children's health, yet up to 48% of children who visit the pediatric emergency department (PED) and urgent care setting are exposed to tobacco smoke. The incorporation of clinical decision support systems (CDSS) into the electronic health records (EHR) of PED patients may improve the rates of screening and brief TSE intervention of caregivers and result in decreased TSE in children.

**Objective:**

We propose a study that will be the first to develop and evaluate the integration of a CDSS for Registered Nurses (RNs) into the EHR of pediatric patients to facilitate the identification of caregivers who smoke and the delivery of TSE interventions to caregivers in the urgent care setting.

**Methods:**

We will conduct a two-phase project to develop, refine, and integrate an evidence-based CDSS into the pediatric urgent care setting. RNs will provide input on program content, function, and design. In Phase I, we will develop a CDSS with prompts to: (1) ASK about child TSE and caregiver smoking, (2) use a software program, Research Electronic Data Capture (REDCap), to ADVISE caregivers to reduce their child's TSE via total smoking home and car bans and quitting smoking, and (3) ASSESS their interest in quitting and ASSIST caregivers to quit by directly connecting them to their choice of free cessation resources (eg, Quitline, SmokefreeTXT, or SmokefreeGOV) during the urgent care visit. We will create reports to provide feedback to RNs on their TSE counseling behaviors. In Phase II, we will conduct a 3-month feasibility trial to test the results of implementing our CDSS on changes in RNs’ TSE-related behaviors, and child and caregiver outcomes.

**Results:**

This trial is currently underway with funding support from the National Institutes of Health/National Cancer Institute. We have completed Phase I. The CDSS has been developed with input from our advisory panel and RNs, and pilot tested. We are nearing completion of Phase II, in which we are conducting the feasibility trial, analyzing data, and disseminating results.

**Conclusions:**

This project will develop, iteratively refine, integrate, and pilot test the use of an innovative CDSS to prompt RNs to provide TSE reduction and smoking cessation counseling to caregivers who smoke. If successful, this approach will create a sustainable and disseminable model for prompting pediatric practitioners to apply tobacco-related guideline recommendations. This systems-based approach has the potential to reach at least 12 million smokers a year and significantly reduce TSE-related pediatric illnesses and related costs.

## Introduction

### Background

Each year over 480,000 deaths in the United States are attributable to smoking, accounting for one in five deaths, and up to one-half of all smokers die prematurely [ 1]. In addition to the harmful consequences to the smoker, exposure of nonsmokers to tobacco smoke (TSE) is a serious health hazard. TSE is unequivocally harmful to children’s health as evidenced by increased rates of asthma, bronchiolitis, and respiratory infections [ 2- 4]. TSE-related illnesses result in increased Pediatric Emergency Department (PED) visits and hospitalizations [ 5- 8]. In the United States, more than 25.5 million children are treated in PEDs each year [ 9] and our own research has found rates of smoking in caregivers as high as 48% (865/1809) [ 10- 13]. Therefore, as many as 12 million children who visit the PED are exposed to tobacco smoke.

The American Academy of Pediatrics (AAP) considers tobacco use a “pediatric disease” given the pediatric morbidity caused by adult tobacco use and TSE [ 14]. The AAP and the *Clinical Practice Guidelines for Treating Tobacco Use and Dependence*(CPGs) [ 15, 16] provide recommendations on the treatment of adult caregivers who smoke for pediatric practitioners, which includes three components: (1) use of electronic health records (EHR) to document or “ASK” about tobacco use and TSE at each clinical encounter, (2) use of clinical decision support systems (CDSS) to “ADVISE” and provide brief cessation counseling in the clinical setting, and (3) “ASSESS” readiness to change and “ASSIST” all smokers in their efforts to make their homes and cars smoke-free and quit smoking. This approach targets the benefits of quitting on reducing the child’s TSE, and offers the potential to decrease tobacco-related morbidity in both the caregiver and child. This expanded use of the EHR to prompt nurses to treat tobacco dependence has been used in adult settings [ 15, 17, 18], and provides a means to standardize screening and counseling of tobacco users in the PED. However, despite these recommendations and evidence of effectiveness in adult settings, no standardized protocols currently exist in PEDs to guide practitioners in the treatment of adult smokers as recommended by the AAP or the CPGs. Our research has shown that tobacco interventions are feasible and effective in the PED setting [ 10, 13].

This project will develop and test new approaches to increase nurse delivery of TSE and cessation interventions to caregivers who smoke who are not pediatric patients. Because past research has shown that PED nurses do not deliver TSE counseling in a systematic way due to barriers such as lack of training, time, and structured systems in intervening with adults [ 19- 22], we plan to integrate CDSS prompts into the EHR to promote national treatment recommendations for caregivers in the PED in a time-efficient, simple way. We will implement a systems-based strategy that can be widely disseminated, and we will systematically assess tobacco use for all caregivers of PED patients.

For a CDSS to be successful, it must be integrated within the complex environment of the health care setting [ 23]. This technology must dynamically interact with practitioners, patients/parents, and existing health care systems [ 23- 25]. One of the biggest challenges with implementation is clinical workflow integration because if the CDSS tools are too difficult or cumbersome to incorporate into the workflow, they will not be used [ 26- 28]. Thus, it has been recommended that prior to integrating a CDSS into the health care setting, it is crucial to ensure that it first reflects the needs and preferences of the users (eg, nurses), and the organization systems (eg, PED) within which it is going to be implemented [ 25]. The CDSS should then only be introduced into clinical practice after an iterative formative evaluation, usability testing, and pilot field testing, designed to facilitate modifications to the CDSS based on user’s needs and the clinical environment [ 25]. We will adopt this developmental approach and pilot testing strategy in the proposed project.

### Framework and Design Objectives for the Clinical Decision Support System

We will adopt the Chronic Care Model as the framework for our proposed CDSS. This model is a widely disseminated paradigm for redesigning health care systems to be more proactive and focused on keeping people healthy rather than reactively treating preventable conditions [ 29- 32]. Because we are addressing caregiver smoking in the PED setting during the child’s (and not the caregiver’s) acute care visit, this model is particularly appropriate and represents an innovative approach for developing our CDSS. Furthermore, because the elements of the model comprise all of the system approaches recommended for tobacco use, it provides a unifying approach for dealing with multiple behavioral risk factors [ 29, 30]. Using this model as the guide, we will work to ensure positive caregiver-nurse interactions through the use of several systems that start during the PED visit.

The systems that we will use to provide a unifying approach that will facilitate caregiver-nurse interactions during the child’s PED visit include: (1) clinical decision support: we will develop and integrate CDSS reminder prompts based on the CPGs [ 16] that will include “ASK,” “ADVISE,” “ASSESS,” and “ASSIST” (“ARRANGE” and “ASSIST” behaviors will be consolidated under the “ASSIST” prompt) and the AAP recommendations (eg, home and car smoking bans), and provide training using the CPGs as a framework, (2) enhanced delivery: we will clearly define nurse roles and establish accountability for these roles by developing Feedback Reports to inform nurses about their TSE counseling behavior, (3) clinical information: we will create a caregiver registry within the EHR to identify smokers, keep track of who has been provided with TSE counseling, and capture caregiver/patient outcomes (using a convenience sample), (4) personalized cessation resources: we will facilitate a direct, “active” referral to the caregivers’ choice of free, evidence-based cessation program/resources (eg, telephone Quitline (QL), SmokefreeTXT, SmokefreeGOV), and (5) self-management support: we will ensure that caregivers are active participants in their care by providing information on cessation resources and written self-help and motivational materials.

The design objectives will be influenced by a team-oriented workflow of care in the PED and local adaptation of the CPGs and other cessation CDSS that have been integrated with the EHR in adult settings [ 15- 18]. The design will include the following steps: identifying caregivers who smoke using easy-to-use screening prompts, providing an electronic link to the brief counseling advice that nurses should provide to caregivers, and evaluating nurse behavior in the ASK, ADVISE, ASSESS, and ASSIST steps. The CDSS prompts will facilitate screening and counseling reminder systems by integrating four different information systems that are part of the total PED system and clinical research infrastructure considered critical for successful implementation. These four systems are: (1) hospital-wide information systems: an enterprise EHR system, Epic, (2) PED clinical systems: the documentations screens used in the current workflow, (3) clinical research: the Research Electronic Data Capture (REDCap) [ 33] database, and (4) the CPGs for tobacco use: links will be provided to generate an active referral to cessation resources based on caregiver preference.

The CDSS will be built using a combination of the EHR and REDCap. The Epic EHR has approximately 11.6% of the total EHR market share in the United States [ 34, 35]. It is used by approximately 835 customers in the United States, securely managing over 1.25 million patient health records per month. Approximately 40% of the US population has its medical information stored in an Epic EHR [ 36]. The emergency department information system allows the addition of modules to existing applications based on customer needs. We will display the child’s TSE and the caregiver’s smoking status within the child’s EHR. However, because the counseling (ie, ADVISE, ASSESS, ASSIST) steps will solely relate to the caregivers, these steps will be completed outside of the child’s EHR in REDCap. The use of REDCap is necessary in order to maintain separate records for the parents/caregivers, who are not patients, and for whom information cannot be stored in the child’s health record. REDCap is a Web-based application for building and managing Internet surveys and longitudinal databases with a secure Web connection with authentication and data logging. It has the ability to create, share, and modify counseling prompt templates for use in any ED setting, can link to external webpages (eg, smokefreeTXT) and has automated, seamless export procedures for data downloads to statistical packages [ 33]. Each PED room contains an Internet-connected computer and access to the EHR. The EHR can provide a prompt to link to REDCap after the caregiver is identified as a current smoker so that the nurse will be able to collect data on caregivers, provide caregivers with cessation counseling messages based on the prompts, obtain QL fax referrals and/or TSE reduction tips, or provide “active” referrals by connecting to the SmokefreeTXT or SmokefreeGOV website [ 33]. REDCap can be accessed at any time so that counseling can be performed and recorded in the patient’s room at the nurse’s convenience. By using existing nursing workflow and automatic prompts, we will create a sustainable intervention requiring minimal additional data entry by nurses, and this will facilitate the clinical workflow between the PED nurse and physician.

### Phase I: Development and Programming of the Decision Support System and Feedback Reports

We will conduct a two phase project with the following aims: Aim 1: convene an advisory panel of PED and EHR technical experts to develop the prototype CDSS with the ASK, ADVISE, ASSESS, and ASSIST prompts to facilitate caregiver tobacco screening and TSE counseling. The CDSS will use a software program (REDCap) that, due to being Web-based, has the capability to interface with an EHR system to trigger brief TSE counseling tips and direct links for registered nurses (RNs) to provide to caregivers. The CDSS template will contain all the necessary elements of text, graphics, and interactive features to ensure generalizability and disseminability. Aim 2: develop feedback reports from data extracted from the CDSS to show individual and overall RN compliance with the use of the tobacco screening and TSE counseling prompts. Aim 3: to refine CDSS functionality and improve feedback reports by conducting RN focus and user groups.

### Phase II: Feasibility and Acceptability Trial of the Decision Support System

Finally, Aim 4: to conduct a feasibility and acceptability trial of the CDSS and feedback reports. We will: (1) obtain a baseline assessment of RNs’ attitudes, barriers, and tobacco-related screening, advising, and assisting behaviors, (2) train RNs on the use of the CDSS and evidence-based TSE counseling strategies and resources for on-going tobacco cessation treatment, (3) assess changes in RNs’ attitudes, perceived barriers, and behaviors 1- and 3-months after training and obtain RN’s satisfaction with the CDSS, (4) assess the smoking behavior and child TSE of a convenience sample of caregivers and children who are current smokers. We will assess changes in TSE-related outcomes of total home/car smoking bans (validated via salivary cotinine) and caregiver smoking behavior (validated by expired CO in those caregivers who report abstinence) in a 10% subset of participants, and (5) conduct exit focused interviews of nurses at 3 months post-training to obtain recommendations for refining and sustaining the CDSS over time.

The following hypothesis will be explored during Phase I and Phase II. Hypothesis 1: at follow-up, there will be an increase in self-reported TSE counseling behaviors and attitudes and decreases in perceived barriers toward TSE counseling by trained RNs. Hypothesis 2: over the 3-month observation period, there will be an increase in CDSS verified TSE counseling behaviors by trained RNs receiving feedback reports. Hypothesis 3: over the 3-month period, there will be lower child TSE and caregivers will report decreases in cigarette use, increased motivation to quit, and increased use of cessation resources compared with baseline.

## Methods

### A Two-Phase Study

**Figure 1:**
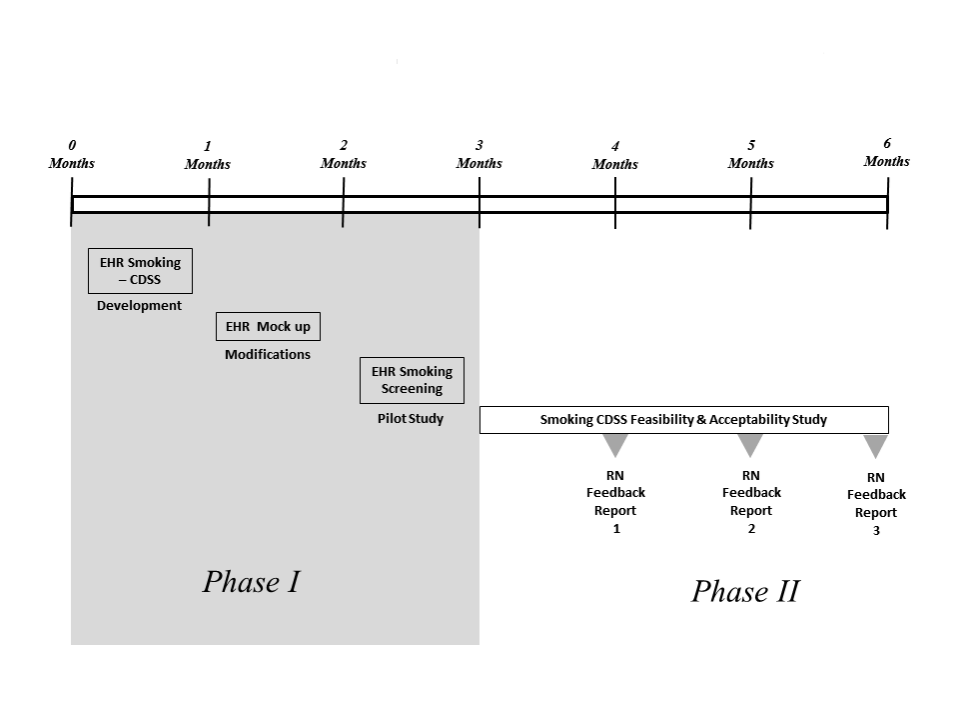
Steps in the development and testing of the smoking Clinical Decision Support System.

This study will be conducted in two phases. In Phase I, we will develop an alpha version of the CDSS with an advisory panel; the alpha version will be revised based on feedback from nurses in focused interviews and small focus groups; this refined version will be iteratively evaluated in a test environment. A portion of the alpha CDSS will be tested in the live environment prior to launching the beta (prototype) full CDSS version. Additionally, we will create nurse feedback reports to encourage the use of the CDSS prompts. The feedback reports will be designed to illustrate how frequently the nurses perform the different tasks within the CDSS compared with other study nurses. In Phase II, we will test the feasibility and acceptability of the CDSS and the feedback reports with RNs working in the Urgent Care portion of the Cincinnati Children’s Hospital Medical Center (CCHMC) PED. Please see Figure 1. Descriptions of each phase are listed below.

### Phase I: Definition Phase and Iterative Usability and Validity Testing

This phase will be devoted to the development, programming, and integration of the CDSS prompts into the EHR and creation of nurse feedback reports. The prompts will facilitate nurse documentation and administration of the screening and TSE counseling components. After developing the CDSS and feedback reports based on input from a multidisciplinary advisory panel, we will conduct nurse-focused interviews, small focus groups, and small user groups to refine the CDSS and feedback report content and interface.

### Setting

CCHMC is the primary provider of inpatient, subspecialty, and emergency care in Greater Cincinnati and the surrounding counties serving over 2 million people. CCHMC is a 628-bed, freestanding, academic, pediatric medical center with more than 1.2 million patient encounters annually and over 800 faculty members and 15,000 employees. It has over 30,000 admissions, 33,000 surgeries, 900,000 ambulatory encounters, and 125,000 emergency department visits per year. This study will occur in the Urgent Care portion of the PED at CCHMC. The PED is a 24/7 facility with five separate Urgent Care sites. The Urgent Care sites are open 7 days a week with varying hours that range from 3 PM to midnight during weekdays and 9 AM to midnight on the weekends. CCHMC uses an enterprise EHR system from Epic. The EHR has been in use since 2009.

### Participants

The participants will consist of an advisory panel and focused interviews and user groups **.**The advisory panel will include a multidisciplinary panel consisting of experts in tobacco cessation, biomedical informatics, PED nurse managers, clinical PED nurses, PED physicians, EHR analysts, and PED flow experts. The focused interviews and user groups will include nurses who work at least once a month in the urgent care will be recruited and verbally consented. These RNs care for 10 to 40 patients during a 12-hour shift. We will obtain institutional review board approval for all study-related activities and consent procedures.

### Procedures for Data Collection and Analysis

The advisory panel will evaluate the current PED workflow and EHR. They will evaluate which providers input information and how data from past records are incorporated into new PED visits. This group will then develop the “ASK, ADVISE, ASSESS, and ASSIST prompts that will be used to identify and counsel smokers and the group will assess how these prompts will affect patient flow and health care delivery to avoid any interruptions in care of the pediatric patient. The CDSS will use REDCap to trigger brief TSE counseling tips and direct links for RNs to provide to caregivers. The counseling steps will not be part of the child’s EHR.

We will conduct focused interviews and small focus groups with PED nurses. During the focus interviews/groups, nurses will be presented with paper copies of mock-ups of each of the prompts in the CDSS and they will provide input on each of the prompts and on the interface with the CDSS in the EHR and with REDCap. They will provide input to make iterative changes. Nurses will be asked structured questions on the content, appearance, design, and format of the prompts and feedback reports and will provide input on the functionality, use, and adaptation of the prompts into their clinical workflow.

After iterative changes have been made based on the feedback provided by the nurses in the focused interviews/groups, we will user-test the CDSS in a test environment to allow nurses to access the CDSS to assess usability. Staff will perform usability testing of the additions to the EHR using observational and “think-aloud” procedures. Nurses will be observed interacting with the CDSS and will be asked to complete a brief survey upon completion of the suggested tasks; any problems will be noted. Notes will be reviewed and the survey will be completed by nurses to determine which independent thematic aspects will be organized around the CDSS and prompts to assess the following: functionality, content, number of “clicks,” length, appearance, format, and type, ease of use, time to use, linkage from Epic to REDCap, linkage to cessation materials, linkage to the SmokefreeGOV website, SmokefreeTXT, integration with regular PED and Urgent Care workflow, technical support and training required, perception of maintenance of use and sustainability, and acceptability of feedback reports. This data will be used to iteratively refine the CDSS and feedback reports for use in Phase II. We will perform an iterative design process. All data will be analyzed, integrated, and used to refine the prompts with components from the CPGs and prior efficacious TSE and cessation interventions [ 10, 13, 16, 37- 39]. Following these analyses, we will develop a list of design changes that will be incorporated into the revised CDSS by the Epic team and used in the next round of usability testing.

### Programming of the CDSS and Creation of Feedback Reports

#### Electronic Health Record Design

The EHR data collection section will fit into the current nursing workflow. The smoking cessation parent and caregiver screening and counseling will be integrated into the existing EHR. This integration will include coding within the EHR and adherence to the existing workflow. This integration will require a link to an external system (REDCap) to complete screening. We will include an external database to collect data because the cessation screening data is directly related to the parents and caregivers (who are not our patients), and therefore is not appropriate to be stored in the child’s EHR. All code is contained and adheres to the CCHMC customization of the Epic EHR.

Adhering to constraints of the EHR, we will integrate cessation screening questions with branching logic to streamline the collection of demographic smoking status data for caregivers. If any caregiver screens positive for smoking, we will launch an alert requesting the nurse provide cessation counseling. The nurse will be able to close the alert and select an appropriate refusal reason if necessary, click a link that goes directly to a REDCap survey, or create a nursing order as a reminder to perform cessation screening at a time that is more convenient. This order will include a link to the REDCap database.

#### Research Electronic Data Capture Design

The REDCap database will be developed based on advisory panel recommendations for the ASK, ADVISE, ASSESS, and ASSIST steps. Patient medical record number will be used to link parent responses with the patient chart. We will use branching logic to display a minimum survey if the caregivers answer “No” to cessation information. The survey results will be used to drive the nursing feedback reports.

#### Feedback Report Design

The nursing feedback reports will be based on the proportion of patients seen during the prior month’s shifts to account for differences in nursing workloads. Feedback reports will display the number of patient caregivers screened (EHR documentation complete in the “ASK” step) compared with the number of patients that the nurse treated that month. For the caregivers screened as positive, REDCap data will be aligned to calculate the proportion of patients who had the ADVISE, ASSESS, and ASSIST steps completed. These data will be presented graphically to the participating nursing staff monthly through a study email account. Nurses will only have their specific results compared with an aggregate average.

### CDSS Screening and Counseling Prompts and Feedback Reports

#### Screening, or ASK Prompt

The caregiver’s current smoking status will be assessed within the EHR, as screening will be integrated into the existing PED workflow during the routine medical intake process by the nurse. The new field will read “Do any of the primary caregivers smoke inside or outside of the home?” If the caregiver answers “Yes,” then a series of question will assess the number of caregivers who smoke, the relationship of the caregivers to the patient, and then finally, if that caregiver smokes “every day” or “some days”. The appropriate smoking status will be selected and recorded within the EHR. If more than one caregiver is present and verified as primary caregivers, both will be screened. If a caregiver has a repeat visit, the prior screening information will be available from the last visit. Completion of the screening prompt will be optional but strongly encouraged for the nurse to proceed further in the patient’s EHR documentation.

Once these questions are completed and one or more caregivers are identified as current smokers, a Best Practice Advisory, pop-up alert window opens up that recommends that smoking cessation counseling be given and asks if the nurse would like to provide counseling. Nurses may select check boxes to counsel now, counsel later (in which case orders will be generated), or to decline counseling for reasons such as patient acuity, time constraints, non-English speaking caregivers, and so on. The completion of the counseling prompts will not be mandatory so that the nurse can use their clinical judgment to opt out as necessary.

Counseling Prompts – Overview

When a caregiver(s) is identified as being a current smoker, the coded results from this electronic question will be used to generate counseling prompts and reminders of the stepwise series of the CPGs. To make these prompts and reminders appear less burdensome, we will consolidate the key counseling elements of the CPGs into three steps: ADVISE to quit, ASSESS readiness to quit, and ASSIST in cessation attempt. Our approach is similar to “Ask, Advise, Refer,” which has been used successfully in other settings [ 40].

ADVISE Prompt

The ADVISE prompt will assist nurses to provide information on how quitting will benefit their child. Nurses will be given training on additional advice that they can give such as: implementing complete home/car smoking bans and quitting smoking; asking caregivers about the perceived impact of smoking on their childÃ¢â‚¬â„¢s health, barriers to implementing smoking bans and/or quitting, and perceived health/lifestyle benefits associated with bans/quitting for their child and themselves; and setting goals for reducing TSE/quitting [ 41, 42].

ASSESS Prompt

The ASSESS prompt will cue the nurse to ask if the caregiver is ready to quit smoking in the next 30 days. Caregivers who respond that they are not ready will be told “I understand that you’re not ready to quit now” and offered an information packet for when they are ready to quit. Those who respond that they are ready or may be ready to quit will be offered assistance by the nurse.

ASSIST Prompt

When the ASSIST prompt is selected, a set of options with branched logic will appear. Nurses will be cued to ask “May I tell you about three options?” The nurses will then be prompted to provide information about one or more of the following options: the QL: Nurses will give information about the services that the QL provides and offered a fax referral to the QL [ 43]; SmokefreeGOV website: nurses will give information about the services that SmokefreeGOV provides and they may log directly onto SmokefreeGOV from REDCap using the computer in the patient’s PED room; smokefreeTXT: nurses will give information about the services that SmokefreeTXT provides and they may log directly onto the SmokefreeTXT site from REDCap where they can fill in the caregiver’s information to sign him/her up for text messages. Finally, caregivers will be offered a packet of written materials [ 40, 44], with information about the effects of smoke exposure on children, the benefits of quitting on their child’s health, how to implement smokefree bans, information on quitting, and information on the QL, the SmokefreeGOV website, and SmokefreeTXT.

Feedback Reports

The use of the CDSS prompts will be further encouraged via feedback reports. Confidential, individualized reports will be provided monthly to each study nurse, similar to the work by Bentz et al [ 17]. For each nurse, we will compute adherence to each of the intervention behaviors (eg, ADVISE, ASSESS, ASSIST) and present these monthly in a feedback report. Each nurse’s feedback report will include a comparison of individual performance to the overall performance average of all of the study RNs [ 17, 45]. Only the study team will have access to the reports, and each nurse will have access only to their report. The study team will introduce the nurses to their first feedback report. Subsequent reports will be delivered monthly (3 total) to each nurse via their personal email; feedback reports will not be used to judge clinical performance.

### Screening Pilot Phase

We will conduct a pilot phase for a period of 2 months in which the nurses will use only the screening questions in the EHR. These will include some or all of the items that ask if any of the primary caregivers smoke, the number of caregivers that smoke, the relationship of the caregivers who smoke to the patient (eg, mother, father, grandmother), and whether those caregivers smoke every day or some days. We will assess the use of these prompts (eg, how often the prompt is used, how many caregivers who smoke are identified) by nurses, and we will email and ask nurses if they experienced any issues or problems while using the screening prompts and/or have any questions.

### Phase II: Feasibility and Acceptability Trial of the Decision Support System

In Phase II, we will conduct a feasibility trial of the CDSS. They will be assessed at baseline, 1 and 3 months after training. The baseline assessment will consist of self-report assessments of TSE counseling behaviors, attitudes, and perceived barriers. In addition, we will enroll a convenience sample of caregivers and their children and assess caregiversÃ¢â‚¬â„¢ smoking behavior and child TSE. We will conduct exit focused interviews with nurses to obtain recommendations on improving the CDSS and perceived barriers to sustainability.

### Setting

Nurses, Caregivers, and Children

Nurses who work at least once a month in the Urgent Care will be recruited and written informed consent will be obtained for participation in the feasibility trial. The clinical research coordinator (CRC) will recruit a convenience sample of 150 caregivers who present to the Urgent Care, are screened, and identified as a smoker who smokes “everyday” or “some days.” The CRC will approach them to obtain informed consent to the collection of baseline and follow-up data about their smoking behavior and their child’s TSE. We will validate child TSE on a racial/ethnically representative sample of children under age 6 at baseline and 3 months, as these children are more likely to be exposed to smoke [ 1- 4].

### Procedures for Feasibility Trial

We will conduct a feasibility and acceptability trial of the CDSS and feedback reports. We will (1) obtain a baseline assessment of nurses’ attitudes, barriers, and tobacco-related screening, advising, and assisting behaviors, (2) train nurses on the use of the CDSS and evidence-based TSE counseling strategies and resources for on-going tobacco cessation treatment, (3) assess changes in attitudes, perceived barriers, and behaviors 1 and 3 months after training and obtain nurses’ satisfaction with the CDSS, (4) assess the smoking behavior and child TSE of a convenience sample of caregivers and children who are current smokers. We will assess changes in TSE-related outcomes of total home/car smoking bans (validated via salivary cotinine) and caregiver smoking behavior (validated by expired CO in those caregivers who report abstinence) in a 10% subset of participants, and (5) conduct exit focused interviews of nurses at 3 months post-training to obtain recommendations for refining and sustaining the CDSS over time.

Our hypotheses are that at 3-month follow-up, there will be an increase in self-reported TSE counseling behaviors and attitudes and decreases in perceived barriers toward TSE counseling by trained RNs and that over the 3-month observation period, there will be an increase in CDSS verified TSE counseling behaviors by trained nurses receiving feedback reports. We also hypothesize that over the 3-month period, there will be lower child TSE and caregivers will report decreases in cigarette use, increased motivation to quit, and increased use of cessation resources compared with baseline.

### Training for Participating Nurses

Prior to the launch of the CDSS in Epic and the start of the feasibility trial, nurses must complete a 20-minute Web-based audiovisual training created by the study investigators. The training will provide an introduction on the importance and effectiveness of TSE counseling and tobacco dependence treatment, and instruction in the use of the CDSS, including: completion of the prompts, delivery of brief TSE and smoking cessation counseling, and how to provide connection with the QL, the SmokefreeGOV website, or SmokefreeTXT during the PED visit [ 46, 47]. During and after the training period, study staff will be available to answer any questions and provide help as needed.

### Baseline Assessments

Nurses

Prior to training, nurses will complete a baseline self-reported assessment to determine their TSE behaviors and attitudes, perception of barriers that inhibit regular intervention with caregivers, and their self-efficacy in intervening with caregivers who smoke. Using principal components analysis, we have developed scales to measure barriers, confidence, and self-efficacy in providing the ASK, ADVISE, ASSESS, and ASSIST behaviors [ 48, 49]. The CRC will conduct an EHR review on a random sample of patients cared for by the Phase II study nurses over the 1-month period prior to training to assess the TSE-reduction behaviors of nurses.

Caregivers and Their Children

We will assess and define the smoking status of all caregivers (eg, current every day smoker, current some day smoker) using the CDSS prompts. The data from the nurses’ counseling prompts (eg, ADVISE and ASSIST) will be summarized.

We will collect detailed in-person smoking behavior data (eg, nicotine dependence, number of cigarettes smoked daily, motivation to quit) on a convenience sample of 150 caregivers and detailed TSE data (eg, number of smokers in the home; presence of smoking bans in the home or car) on their children. We will conduct cotinine analyses of child saliva samples collected on a sample of children at baseline to validate child TSE [ 2- 4].

### Follow-up Assessments

#### Self-Report by Nurses

Nurses will be reassessed by self-report at 1 and 3 months after training to evaluate change in practice behaviors, attitudes, barriers, and self-efficacy in intervening with caregivers and providing screening and TSE counseling, and maintenance of the intervention components. We will use the same scales as used at baseline to measure their intervention-related barriers, confidence, and self-efficacy.

#### Exit Focused Interviews

After the 3-month assessment, the study team will conduct exit focused interviews in which nurses will be asked for suggestions on how to improve the CDSS and they will be asked to identify factors and barriers that they perceive will be associated with the sustainability of the CDSS components into real-world practice. Data will be analyzed and results and recommendations incorporated into the design of a future, large R01 trial.

EHR Review of Patients

During the entire 3-month period of the pilot trial, the CRC will conduct daily EHR reviews for each patient cared for by the nurses whose caregiver screened positive for current smoking. The review will assess sociodemographics, chief complaint, and discharge diagnosis. The CRC will assess the nurses’ compliance with the TSE components: Ask about smoking, Advise to quit, Assess readiness to quit, Assist in quitting, and delivery of written materials.

Caregiver Cessation and Child TSE

Smoking behavior and cessation outcomes will be assessed by self-report at 3-months on the convenience sample of 150 caregivers that were assessed at baseline; child TSE will be validated with salivary cotinine on the 10% subsample via in-person visits at 3-months. Outcomes will include nicotine dependence; readiness to change; quit attempts ≥24 hours; nicotine replacement therapy or use of cessation resources, abstinence; and child TSE. Abstinence in caregivers will be verified via expired carbon monoxide testing during in-person visits.

### Analytic Plan

Preliminary Data Analyses

Prior to the primary analyses, we will generate descriptive statistics for all variables (eg, means, medians, standard deviations, ranges, skewness) and we will examine potential outliers and patterns of missing data. To assess potential enrollment bias of nurses and/or caregivers, we will compare demographics of those enrolled versus those not enrolled; differences will be assessed according to variable type, chi-square test/Fisher’s exact test for categorical variables or *t*-tests/Wilcoxon-Mann Whitney tests for continuous variables. Attrition analyses will be done to assess if those nurses without complete follow-up differ from those retained. If key independent variables significantly predict attrition, we will conduct the analysis with and without drop-outs. Primary outcomes will be compared using complete case analysis and intention to treat, assuming drop-outs are like those who do not comply with the TSE counseling behaviors.

#### Analysis of Focused Interview and User Group Data in Phase I

We will develop and integrate the CDSS into the EHR using qualitative data from the advisory panel and focused interview and user group data. We will present the prototype CDSS with the ASK, ADVISE, and ASSIST prompts to the advisory panel and ask for their views on use of the CDSS interface with the child’s EHR. This CDSS will be iteratively edited based on expert advice. We will create the feedback reports and nurse recommendations, which will be based on user group input. Detailed notes will be obtained, typed, and evaluated to determine which independent thematic aspects will be organized around the CDSS and prompts to assess: functionality, content, number of clicks, length, appearance, format, and type, ease of use, time to use, linkage from the EHR to REDCap, linkage to cessation materials, linkage to the SmokefreeGOV website, linkage to SmokefreeTXT, integration with regular PED and Urgent Care workflows, technical support and training required, perception of maintenance of use and sustainability, and acceptability of feedback reports. This data will be used to iteratively refine the CDSS and reports for use in Phase II. Additionally, exit focused interviews at 3 months will be used to obtain nursesÃ¢â‚¬â„¢ views on the factors, barriers, suggestions, and recommendations on improving the CDSS. Data will be synthesized and used in future trials.

Analysis of Data from Feasibility Trial

To assess if there are increased self-reported TSE counseling behaviors (primary outcomes), improved attitudes and decreased barriers toward counseling (secondary outcomes) by study nurses, we will use the self-assessment at baseline, 1, and 3 months and examine changes in reported and EHR-verified behaviors, and changes in attitudes and barriers at baseline and each follow-up point. The first approach will be the intention to treat analysis; a generalized linear mixed-model will be used to allow use of the appropriate link function and examination of change over time. Second, incorporation of generalized estimating equations will allow for missing data, and then addition of any potential covariates.

To assess if there are increased CDSS-based TSE counseling behaviors by nurses receiving feedback reports, we will conduct an EHR review of 100% of the patients cared for by study nurses during the 3 months of the feasibility trial. We will compute descriptive statistics of compliance with the prompts. Options selected under these prompts will include: Ask about smoking, Advise to quit, Assess readiness to quit, and Assist in TSE reduction and quit attempt advice (QL fax, SmokefreeGOV website, SmokefreeTXT, and delivery of written materials). For each nurse, we will compute the percentage of the tobacco cessation intervention behaviors addressed (eg, Advise, Assess, Assist) and obtain descriptive statistics. We will assess if there are trends in the association of nurse protocol to child sociodemographics, chief complaint, or discharge diagnosis. As above, we will employ the generalized linear mixed-modeling to account for the nesting of caregivers within nurses and look at changes over time, adding the potential time-dependent covariates into the model.

Finally, we will assess if there is lower TSE in children and if caregivers will report decreased cigarette use, increased motivation to quit, and increased use of cessation resources at 3 months compared with baseline. Participant characteristics at baseline and 3 months will be summarized using descriptive statistics. Pairwise (within subjects) *t*-tests or McNemar’s test or Cochran’s Q test, as appropriate, will be conducted to examine differences in child TSE and caregiver smoking behavior and motivation to quit at baseline compared with 3 months.

## Results

This study is ongoing. We are currently in the data collection and analysis stage of Phase II. This study may inform future research among pediatric urgent care or emergency department nurses and other providers working with adult caregivers who use tobacco. The results will provide evidence as to whether brief tobacco screening and tobacco cessation counseling prompts are feasible, acceptable, and easily incorporated into the workflow of urgent care nurses.

## Discussion

This is the first study to develop a CDSS, integrate it with a widely-used EHR (Epic), and test its use to facilitate tobacco screening and TSE counseling of adult smokers in the PED setting. The CDSS will use a system of prompts and templates that can be implemented in all emergency settings regardless of the EHR type. We will ensure that the CDSS is reproducible, generalizable, and disseminable by carefully choosing an EHR platform and research template which allows for the creation, sharing and modification of prompt templates regardless of setting (pediatric or adult). Thus, if effective, this type of CDSS and systems-based strategy can be widely disseminated and incorporated into other emergency settings.

By incorporating tobacco screening and treatment into an existing clinical EHR system and providing prompts and feedback on the delivery of TSE and tobacco treatment, we will maximize the probability of creating a sustainable, disseminable model for use in the PED setting. If successful, this research has a high likelihood of translation into other emergency settings and sustainability over time. This proposed approach has the potential to reach over 12 million smokers a year and significantly reduce TSE-related pediatric illness and related costs.

## Abbreviations

AAP: American Academy of Pediatrics
CCHMC: Cincinnati Children’s Hospital Medical Center
CDSS: clinical decision support systems
CPGs: clinical practice guidelines
CRC: clinical research coordinator
EHR: electronic health records
PED: pediatric emergency department
REDCap: Research Electronic Data Capture
RNs: registered nurses
QL: Quitline
TSE: tobacco smoke exposure

## Appendix

Multimedia Appendix 1 NIH study section reviews of this funded project.
