# Supplementary material for: Development and Testing of a Computerized Decision Support System to Facilitate Brief Tobacco Cessation Treatment in the Pediatric Emergency Department: Proposal and Protocol
Source: JMIR Res Protoc. 2016 Apr 20;5(2):e64. doi: 10.2196/resprot.4453 (PMC4856881; doi:10.2196/resprot.4453)
Supplement: Multimedia Appendix 1 [file resprot_v5i2e64_app1.PDF]

SUMMARY STATEMENT
(Privileged Communication)

PROGRAM CONTACT:
Yvonne Hunt
(240) 276-6975
huntym@mail.nih.gov

Release Date: **08/11/2014**

Application Number: **1 R21 CA184337-01A1**

Principal Investigators (Listed Alphabetically):
GORDON, JUDITH S PHD
MAHABEE-GITTENS, E. MELINDA MD (Contact)

Applicant Organization: CINCINNATI CHILDRENS HOSP MED CTR

Review Group: **ZCA1 RTRB-R (O1)**
National Cancer Institute Special Emphasis Panel
Omnibus SEP-1

Meeting Date: **07/29/2014**
Council: **OCT 2014**
Requested Start: **12/01/2014**
RFA/PA: **PAR13-146**
PCC: **Y2TC**

Project Title: **Pediatric Emergency Department Decision Support System to Reduce Secondhand Smoke**
SRG Action: **Impact Score: 23 Percentile: 9 #**
Next Steps: **Visit http://grants.nih.gov/grants/next_steps.htm**
Human Subjects: 30-Human subjects involved - Certified, no SRG concerns
Animal Subjects: 10-No live vertebrate animals involved for competing appl.
Gender: **1A-Both genders, scientifically acceptable**
Minority: **1A-Minorities and non-minorities, scientifically acceptable**
Children: **1A-Both Children and Adults, scientifically acceptable**
NIH Defined Phase III Clinical trial

Project Year	Direct Costs Requested	Estimated Total Cost
1	125,000	200,885
2	150,000	241,062
TOTAL	275,000	441,947

ADMINISTRATIVE BUDGET NOTE: The budget shown is the requested budget and has not been adjusted to reflect any recommendations made by reviewers. If an award is planned, the costs will be calculated by Institute grants management staff based on the recommendations outlined below in the COMMITTEE BUDGET RECOMMENDATIONS section.

RESUME AND SUMMARY OF DISCUSSION: The hypothesis of the applicant is that prompting pediatric practitioners to apply tobacco-related guideline recommendations in the pediatric emergency department (PED) will lead to reduction in tobacco use in care-givers and thereby a reduction in second-hand smoke exposure (SHSe) – related pediatric illness. The applicant proposes to use PED systems to identify smokers for an intervention to address SHSe. Nurses will be engaged to develop, refine, and integrate a decision support system (DSS) to promote cessation counseling in this setting. The intervention will include three parts: asking about child SHSe and care-giver smoking, advising care-givers to reduce their child's SHSe by implementing total smoking home and car bans and quitting smoking, and finally assisting care-givers to quit by connecting them to the Quit-line during the PED visit. Nurses will receive feed-back about their performance and this input will be taken into consideration to refine the program content, functions, and design. Changes in nurse performance on SHSe and related counseling will be assessed in a three-month feasibility study. It is a resubmitted application and the applicant has addressed most of the reviewers' concerns by adding the measurement of salivary cotinine level as a marker of SHS, conducting care-giver tobacco behavior at baseline and cessation outcomes three month after the intervention by using self-report measures and biochemically validation of smoking status, and adding additional information on electronic medical record (EMR) effectiveness, generalizability of DSS and patient flow. Strengths of the application include the significant public health relevance to target SHSe since reduction of SHSe could help reduce the risk for various childhood health issues; the strong preliminary studies of the applicant that increases the feasibility of the proposed project; the innovative use of the PED for smoking cessation intervention of the care-givers that may provide a meaningful and emotionally impactful context to motivate smoking abstinence; the integration of multiple methodological components and procedures in addressing the project aims; the potential for scalability and generalizability to other pediatric settings of this intervention model; the assessment of care-giver smoking-related variables; the well-qualified research team; and the strong research environment, with plenty of resources to conduct the proposed study. However, several minor weaknesses lower the merit of the application. These include the lack of assessment of care-giver response to the intervention; the concern that smoking cessation may take a back-seat to other issues in a PED; the lack to clearly define the evaluation frame-work that links various standard feasibility study criteria, such as reach, effectiveness, acceptability to patients and providers; the lack to exclude families with adolescent smokers; and the lack to prepare the intervention materials in Spanish when the Spanish-speaking communities are tailored. Nevertheless, by developing interventions to reduce SHSe, the project has a high impact in its associated health problems, especially among children.

DESCRIPTION (provided by applicant): Second-hand smoke exposure (SHSe) is unequivocally harmful to children's health as evidenced by increased rates of asthma, bronchiolitis, and respiratory infections. Up to 48% of children who visit the pediatric emergency department (PED) are exposed to SHS. Our research has shown that tobacco interventions are feasible and effective in the PED setting. However, PED nurses (RNs) do not deliver SHSe counseling in a systematic way due to barriers such as lack of training, time, and structured systems. The Clinical Practice Guidelines for Treating Tobacco Use and Dependence (CPGs) recommends that pediatric practitioners treat adult caregivers who smoke in all clinical encounters by using: 1) electronic medical records (EMR) to document tobacco use and SHSe, 2) "prompts" within a decision support system (DSS) to promote cessation counseling and urge all smokers to quit, and 3) advice to all smokers that the only effective protection from SHSe is to make their homes and cars smoke-free. The expanded use of the EMR to prompt RNs to treat tobacco dependence and provide feedback has been used successfully in adult settings, and provides a means to standardize screening and counseling of adult tobacco users in the PED. However, DSS tools designed to facilitate SHSe reduction have not been developed for use in the PED. We propose a study that will be the first to develop and evaluate a PED DSS-EMR System to facilitate the identification of smokers and the delivery of a SHSe intervention to caregivers who bring their child to the PED. We will conduct a two- phased project to develop, refine, and integrate an Epic-based DSS, using the CPGs,

our prior PED cessation work, and our effective web-based cessation training program. In Phase I, we will develop a three-part DSS with prompts to: 1) ASK about child SHSe and caregiver smoking using the same screening prompts required by the Meaningful Use incentive program, 2) Use a free software program (REDCap) to ADVISE caregivers to reduce their child's SHSe via total smoking home and car bans and quitting smoking, and 3) ASSIST caregivers to quit by directly connecting them to their choice of free cessation resources (e.g., Quit- line, txt2quit, smokefree.gov) during the PED visit. We will create reports to provide feedback to RNs on their SHSe counseling behaviors. RNs will provide input on program content, functions, and design. In Phase II, we will conduct a 3-month feasibility trial to test the results of implementing our DSS on changes in RN SHSe-related behaviors, and child and caregiver outcomes. If effective, program results, procedures, and REDCap templates will be disseminated easily. The DSS will use a system of prompts and templates that can be modified, shared and used in all emergency settings (e.g., pediatric or adult), regardless of EMR type. Compliance and interest in the DSS will be increased by incorporating the widely-used Meaningful Use tobacco recording requirements, further increasing relevance to other institutions. This systems-based approach has the potential to reach at least 12 million smokers a year and significantly reduce SHSe-related pediatric illnesses and related costs.

PUBLIC HEALTH RELEVANCE: The prevalence of secondhand smoke exposure among children who visit the pediatric emergency department and tobacco use among their caregivers is high. This project will develop, iteratively refine, integrate, and pilot test the use of an innovative decision support system to prompt pediatric emergency department nurses to provide secondhand smoke exposure reduction and smoking cessation counseling to caregivers who smoke. If successful, this approach will create a sustainable and disseminable model for prompting pediatric practitioners to apply tobacco-related guideline recommendations, thereby reducing secondhand smoke exposure related pediatric illnesses and tobacco use in caregivers.

CRITIQUES: The written critiques of individual reviewers are provided in essentially unedited form in this section. Please note that these critiques and criterion scores were prepared prior to the meeting and may not have been revised subsequent to the discussions at the review meeting. The "Resume and Summary of Discussion" section summarizes the final opinions of the review committee.

CRITIQUE 1:

Significance:	1
Investigator(s):	1
Innovation:	2
Approach:	1
Environment:	1

Overall Impact: In this resubmitted application, the applicant focuses on using pediatric emergency department systems to identify smokers for an intervention to address second-hand smoke. Care-givers who bring their children to the emergency department will be targeted. Nurses will be engaged using an approach to develop, refine, and integrate a decision support system to promote cessation counseling in this setting. The intervention itself will include three parts: asking about child second-hand exposure and care-giver smoking, advising care-givers to reduce their child's second-hand exposure by implementing total smoking home and car bans and quitting smoking, and finally assisting care-givers to quit by connecting them to the Quit-line during the emergency visit. Nurses will receive feed-back about their performance and this input will be taken into consideration to refine program content, functions, and design. Changes in RN performance on second-hand exposure and related counseling will be assessed in a three-month feasibility study. The application is significant as targeting second-

hand smoke exposure is an important public health issue. Reducing exposure to second-hand smoke could help reduce risk for various childhood health problems. Previous work by the Principal Investigator and others serves as a background. The use of the time and place could present an opportunity to curb second-hand smoke among parents. Additional strength include the integration of multiple methodological components and procedures in addressing the study aims.

1. Significance:

Strengths

- The significance of this work is high in light of the adverse health effects of second-hand smoke exposure. Interventions to reduce exposure to second-hand smoke should help reduce associated health problems, especially among children.

Weaknesses

- None noted.

2. Investigator(s):

Strengths

- The team planning this project is well-qualified. The Principal Investigator is a professor of clinical pediatrics, with substantial experience related to the proposed work and population.
- The project builds nicely on what the Principal Investigator has done before and there are excellent resources increasing confidence in successful completion of the proposed research.

Weaknesses

- None noted.

3. Innovation:

Strengths

- Elements in this plan are novel, including the use of a decision support system and the approach to promote counseling for cessation among smoking care-givers. The use of the emergency department setting is important and opportunistic.

Weaknesses

- None noted.

4. Approach:

Strengths

- Well-developed procedures are planned for this project, with a significant goal to promote efforts to reduce second-hand smoking and facilitate smoking cessation among parents.
- The method and procedures take advantage of resources available in the ER setting to promote the identification of smoking care-givers and provide smoking cessation assistance.
- The theoretical base for this work is sound and the hypotheses are clear; the plan provides a meaningful way to test them.
- Focusing the work on nurses in the pediatric emergency room is important and can have potential impact in expanding services in this important clinical setting.

- The work is based on preliminary work by this team, which should facilitate the timely completion of this proposed research.

Weaknesses

- None noted.

5. Environment:

Strengths

- Plenty of resources available to the investigative team to conduct the proposed research.
- Previous research related to the proposed work increases feasibility of the study.

Weaknesses

- None noted.

Protections for Human Subjects: Acceptable Risks and/or Adequate Protections

Data and Safety Monitoring Plan (Applicable for Clinical Trials Only): Not Applicable (No Clinical Trials)

Inclusion of Women, Minorities and Children:

G1A - Both Genders, Acceptable

M1A - Minority and Non-minority, Acceptable

C1A - Children and Adults, Acceptable

- Adequate details are provided about the measures to protect confidentiality.

Resubmission:

- The applicants are responsive to previous reviews. Information about measuring second-hand smoke is added and in 20% of children salivary cotinine will be measured. Care-giver tobacco behavior at baseline and cessation outcomes three-months after the intervention by using self-report measures and biochemically validation of smoking status will be conducted. Additional information on EMR effectiveness, generalizability of DSS, and patient flow is added to address previous review comments.

CRITIQUE 2:

Significance:	1
Investigator(s):	1
Innovation:	1
Approach:	3
Environment:	1

Overall Impact: Second-hand smoke (SHS) exposure is a pervasive and well-documented public health problem. In this innovative project, the applicant proposes to conduct a feasibility study for an EMR Decision Support System (DSS) to reduce SHS among children in a Pediatric ED (PED) setting.

EMR algorithms will prompt practitioners to offer screening and smoking cessation counseling to parents/care-givers using tailored patient materials and provider feed-back reports. The application has high potential impact because of the public health importance of the topic; the carefully-conceived approach; the expertise of the investigators; the suitability of the research environment; and, if successful, the likelihood that it will provide data necessary for future research with the ultimate goal of reducing the harmful consequences of tobacco use among parents and SHS exposure among their children.

1. Significance:

Strengths

- This proposed work is significant because it addresses a critical public health problem – exposure to second-hand smoke among children. This is a major strength.
- The use of the PED setting is a major strength because it may provide a meaningful and emotionally impactful context to motivate smoking abstinence among care-givers.
- The applicant's approach allows for the potential for scalability and generalizability to other pediatric settings, a major strength.

Weaknesses

- None noted

2. Investigator(s):

Strengths

- The investigative team has the necessary complement of skills, experience, and collaborative history to effectively implement the proposed study. These factors include developing and implementing DDS, knowledge of pediatrics and the PED setting, clinic-based smoking cessation intervention research, ecological momentary assessment, clinical information systems, and qualitative and quantitative data analysis. This is a major strength.
- A major strength is the long-term collaboration between the Principal Investigator (Dr. Mahabee-Gittens) and Dr. Gordon, a well-established, experienced investigator.

Weaknesses

- None noted.

3. Innovation:

Strengths

- A major strength of this study is its innovation. It represents a pioneering effort to develop a DSS that is integrated with the widely-used Epic EMR platform and then test its feasibility to guide tobacco-use screening and smoking cessation in a pediatric ED setting.
- The use of practitioner engagement throughout the entire project to help ensure that project methods are feasible and acceptable in the clinic setting is a major strength.

Weaknesses

- None noted

4. Approach:

Strengths

- Preliminary studies are strong and support the experience of the research team and the feasibility of the study approach.
- The study will begin by convening an advisory panel of PED and EMR experts to develop the DDS. The investigators will continue to solicit feed-back from practitioners, e.g., RN focus groups. This engagement is a major strength because it provides a protocol for developing a site-relevant DDS, thereby the likelihood that such an approach will be adopted and implemented in other settings.
- The study hypotheses are clear and appropriate based on previous research.
- The use of the Chronic Care Model and the 5 A's frame-work provide a solid theoretical and evidence-based basis for the DDS.
- Provider roles are clearly-defined, training and incentives are appropriate, and feed-back is provided.
- The use of Epic and REDCap is a strength since these are widely-used systems.
- From one perspective, the use of specific setting and methods (e.g., CCH, Epic) limits the generalizability of the study. However, the engagement of an advisory panel and the opportunity for ongoing feed-back from RNs are methods that are transferable to other settings with other EMR systems.
- Assessment of care-giver smoking-related variables is another strength.

Weaknesses

- There is no assessment of care-giver response to the intervention.
- Investigators could have more fully addressed the concern that smoking cessation will take a back-seat to other issues in an ED.
- There is no clearly-defined Evaluation Frame-work that links the various assessments to standard feasibility study criteria, e.g., reach, effectiveness, acceptability to patients and providers, etc.

5. Environment:

Strengths

- The Cincinnati Children's Hospital and Medical Center constitute an outstanding environment for the proposed study. The letters of support indicate strong enthusiasm for the project.

Weaknesses

- None noted.

Protections for Human Subjects: Acceptable Risks and/or Adequate Protections

- Risks and protections are adequately explained.

Data and Safety Monitoring Plan (Applicable for Clinical Trials Only): Acceptable

- The DSMP is adequately described and acceptable.

Inclusion of Women, Minorities and Children:

G1A - Both Genders, Acceptable

M1A - Minority and Non-minority, Acceptable

C1A - Children and Adults, Acceptable

- Children's involvement is restricted to EMR review and saliva cotinine collection (20% of the sample). Children over 10 will complete an Assent Form.

Biohazards: Acceptable

Resubmission: The investigators responded effectively the reviewer feedback.

Resource Sharing Plans: Acceptable

Budget and Period of Support: Recommend as Requested

CRITIQUE 3:

Significance:	2
Investigator(s):	1
Innovation:	2
Approach:	3
Environment:	2

Overall Impact: The investigators propose a careful development and study of an intervention tool to be used in a large children's hospital emergency room, which will allow EMR-guided, nurse-led intervention to reduce second-hand smoking. The applicants have prepared a well-thought out plan of how to develop this intervention and how to test its feasibility. The co-Principal Investigators have significant experience in this field and have gathered a team of experts to insure the success of the project. The intervention, once developed and tested, will lead to a future application to test the efficacy of reduction of second-hand smoke exposure in children.

1. Significance:

Strengths

- The study has the potential to create new standards of assessment and intervention regarding care-taker smoking and the applicant addresses "Meaningful Use" mandates to record and address smoking behavior in a systematic way.
- The work is significant in the possibility that it will be useful in reducing pediatric exposure to second-hand smoke.
- Development of a system that uses EPIC EMR and RedCap will allow for dissemination, if successful, and is the kind of research that the use of electronic medical records and decision support programs should be promoting.

Weaknesses

- Pediatric patients are often seen in general emergency rooms, so that smoking interventions are more often tailored for adults, not parents, and not for second-hand smoke. Generalizability may be limited to large children's hospital emergency rooms.

- The investigators cite that three million visits to pediatric emergency rooms are due to SHSe, but they do not target their intervention to children whose presenting symptom is due related to SHSe.

2. Investigator(s):

Strengths

- The team of investigators is superb, with significant experience and success in this type of study.
- Creation of a team with complementary talents and experience is commendable.

Weaknesses

- None noted

3. Innovation:

Strengths

- The combination of EMR-based decision support, nurse training for intervention, use of parent technology for quit support are all innovative components of this study.
- The use of a “teachable moment” visit in which the potential health of a child is related to a negative behavior in a parent is innovative.

Weaknesses

- None noted

4. Approach:

Strengths

- The use of a multi-disciplinary advisory panel will allow for development of an intervention that includes both innovative EMR use and an understanding of patient flow in the ED.
- The addition, in the resubmitted application, the measures of change in SHSe at baseline and at three months is an important change. This will include salivary cotinine as a potential marker of exposure.
- The investigators appear to have constructed a rigorous plan for intervention development, testing, RN training, and assessment.
- The intervention the investigators will develop, refine, and test for feasibility will provide data and a frame-work for a future R01.

Weaknesses

- How will you know that the exposure measured in salivary fluid is related to the subject (parent) in the ED using tobacco as opposed to other care-taker and community exposures, or in the case of adolescent smokers, smoking themselves?
- Families with adolescent smokers (where the patient in the ED is an adolescent who smokes) should be excluded. The issue of SHSe in these teenagers is less important than the issue of their smoking behavior in general. A stronger study would be in parents of children with respiratory illnesses, where SHS is an important negative impact on child health. This would seem more of an appropriate use of time/resources in a pediatric emergency room.

- Preparation of materials and interventions in Spanish would greatly help this project. Tailoring to Spanish-speaking communities, as noted in the resubmitted application, is admirable, but delays the potential to learn for the design of an R01, which should include Spanish speakers.

5. Environment:

Strengths

- Cincinnati Children's Hospital and Medical Center are an excellent environment for conduct of this study. The support of IT, the ED, and the nursing department are clearly available.

Weaknesses

- None noted.

Protections for Human Subjects: Acceptable Risks and/or Adequate Protections

- The subjects will participate in an IRB-approved survey.

Data and Safety Monitoring Plan (Applicable for Clinical Trials Only): Acceptable

  - The PI elucidates plans for monitoring by the study team and annual review for the study overall.

Inclusion of Women, Minorities and Children:

G1A - Both Genders, Acceptable

M1A - Minority and Non-minority, Acceptable

C3A - No Children Included, Acceptable

Resource Sharing Plans: Acceptable

Budget and Period of Support: Recommend as Requested

THE FOLLOWING RESUME SECTIONS WERE PREPARED BY THE SCIENTIFIC REVIEW OFFICER TO SUMMARIZE THE OUTCOME OF DISCUSSIONS OF THE REVIEW COMMITTEE ON THE FOLLOWING ISSUES:

PROTECTION OF HUMAN SUBJECTS (Resume): ACCEPTABLE
Human subjects' protections are well-described and an acceptable DSMP is in place.

INCLUSION OF WOMEN PLAN (Resume): ACCEPTABLE G1A
Both genders are included.

INCLUSION OF MINORITIES PLAN (Resume): ACCEPTABLE M1A
Minorities are included.

INCLUSION OF CHILDREN PLAN (Resume): ACCEPTABLE C1A

Children are included. Children's involvement is restricted to EMR review and saliva cotinine collection (20% of the sample). Children over 10 will complete an Assent Form.

COMMITTEE BUDGET RECOMMENDATIONS: The budget was recommended as requested

---

Ad hoc or special section application percentiled against "Total CSR" base.

NIH has modified its policy regarding the receipt of resubmissions (amended applications). See Guide Notice NOT-OD-14-074 at http://grants.nih.gov/grants/guide/notice-files/NOT-OD-14-074.html. The impact/priority score is calculated after discussion of an application by averaging the overall scores (1-9) given by all voting reviewers on the committee and multiplying by 10. The criterion scores are submitted prior to the meeting by the individual reviewers assigned to an application, and are not discussed specifically at the review meeting or calculated into the overall impact score. Some applications also receive a percentile ranking. For details on the review process, see http://grants.nih.gov/grants/peer_review_process.htm#scoring.

MEETING ROSTER

National Cancer Institute Special Emphasis Panel
NATIONAL CANCER INSTITUTE
Omnibus SEP-1
ZCA1 RTRB-R (O1) S
July 29, 2014 - July 30, 2014

CHAIRPERSON

DIGNAN, MARK B., PHD
PROFESSOR
DEPARTMENT OF INTERNAL MEDICINE
PREVENTION RESEARCH CENTER
MARKEY CANCER CENTER
UNIVERSITY OF KENTUCKY
LEXINGTON, KY 40536

MEMBERS

AL'ABSI, MUSTAFA N, PHD
PROFESSOR AND DIRECTOR
DEPARTMENT OF BIOBEHAVIORAL HEALTH
AND POPULATION SCIENCES
UNIVERSITY OF MINNESOTA MEDICAL SCHOOL
DULUTH, MN 55812

ARNOLD, CONNIE L, PHD
PROFESSOR
DEPARTMENT OF MEDICINE
AND PEDIATRICS
HEALTH SCIENCES CENTER
LOUISIANA STATE UNIVERSITY
SHREVEPORT, LA 71103

BLOOM, JOAN R, PHD
PROFESSOR
DEPARTMENT OF HEALTH POLICY AND MANAGEMENT
UNIVERSITY OF CALIFORNIA, BERKELEY
BERKELEY, CA 94720

BRADEN, CARRIE JO , PHD
ASSOCIATE DEAN FOR RESEARCH
DEPARTMENT OF CHRONIC NURSING CARE
SCHOOL OF NURSING
UNIVERSITY OF TEXAS HEALTH SCIENCES CENTER
SAN ANTONIO, TX 78229

BRANDT, HEATHER M, PHD
ASSOCIATE PROFESSOR
HEALTH PROMOTION, EDUCATION & BEHAVIOR
CORE FACULTY, CANCER PREVENTION & CONTROL
PROGRAM
ARNOLD SCHOOL OF PUBLIC HEALTH
UNIVERSITY OF SOUTH CAROLINA
COLUMBIA, SC 29208

COOPER, GREGORY S., MD
PROFESSOR OF MEDICINE
DEPARTMENT OF MEDICINE
CASE WESTERN RESERVE UNIVERSITY
CLEVELAND, OH 441065066

CRONAN, THEREASA A, PHD
PROFESSOR
DEPARTMENT OF PSYCHOLOGY
COLLEGE OF SCIENCES
SAN DIEGO STATE UNIVERSITY
SAN DIEGO, CA 92120

D'ONOFRIO, CAROL N, DPH, DRPH
PROFESSOR EMERITA
DEPARTMENT OF PUBLIC HEALTH
SCHOOL OF PUBLIC HEALTH
UNIVERSITY OF CALIFORNIA BERKELEY
PIEDMONT, CA 94611

DAHIYA, RAJVIR , PHD
PROFESSOR AND DIRECTOR
DEPARTMENT OF UROLOGY
VETERAN AFFAIRS MEDICAL CENTER
UNIVERSITY OF CALIFORNIA, SAN FRANCISCO
SAN FRANCISCO, CA 94143

DALY, MARY BERYL, MD, PHD
ASSOCIATE DIRECTOR
CANCER CONTROL SCIENCE PROGRAM
FOX CHASE CANCER CENTER
TEMPLE UNIVERSITY HEALTH SYSTEM
PHILADELPHIA, PA 19111

DILLER, LISA R., MD
PROFESSOR OF PEDIATRICS
DEPARTMENT OF ONCOLOGY
DANA-FARBER CANCER INSTITUTE
HARVARD UNIVERSITY
BOSTON, MA 02115

DINO, GERI A., PHD
PROFESSOR AND DIRECTOR
WEST VIRGINIA PREVENTION RESEARCH CENTER
DEPARTMENT OF COMMUNITY MEDICINE
WEST VIRGINIA UNIVERSITY
MORGANTOWN, WV 265069190

DJURIC, ZORA , PHD
PROFESSOR
DEPARTMENT OF FAMILY MEDICINE
CANCER AND GERIATRICS CENTER
UNIVERSITY OF MICHIGAN
ANN ARBOR, MI 48109

DORGAN, JOANNE F, PHD
PROFESSOR
DEPARTMENT OF EPIDEMIOLOGY AND PUBLIC HEALTH
SCHOOL OF MEDICINE
UNIVERSITY OF MARYLAND
BALTIMORE, MD 21201

FERNANDER, ANITA F, PHD
ASSOCIATE PROFESSOR, DIRECTOR OF GRADUATE
STUDIES
DEPARTMENT OF BEHAVIORAL SCIENCE
COLLEGE OF MEDICINE
UNIVERSITY OF KENTUCKY
LEXINGTON, KY 40536

FISHER, SUSAN G, PHD
PROFESSOR AND CHAIR
DEPARTMENT OF CLINICAL SCIENCES
TEMPLE UNIVERSITY
PHILADELPHIA, PA 19140

GOLD, ELLEN B., PHD
PROFESSOR AND CHAIR
DEPARTMENT OF PUBLIC HEALTH SCIENCES
UNIVERSITY OF CALIFORNIA AT DAVIS
DAVIS, CA 95616

HU, JENNIFER J., PHD
PROFESSOR, ASSOCIATE DIRECTOR FOR CANCER
PREVENTION CONTROL
DEPARTMENT OF EPIDEMIOLOGY & PUBLIC HEALTH
SYLVESTER COMPREHENSIVE CANCER CENTER
UNIVERSITY OF MIAMI MILLER SCHOOL OF MEDICINE
MIAMI, FL 33136

JEFFE, DONNA B, PHD
PROFESSOR
DIVISION OF HEALTH BEHAVIOR
DEPARTMENT OF MEDICINE
SCHOOL OF MEDICINE
WASHINGTON UNIVERSITY
ST. LOUIS, MO 63108

KELLEY, WILLIAM , PHD
ASSOCIATE PROFESSOR
DEPARTMENT OF PSYCHOLOGICAL AND BRAIN
SCIENCES
DARTMOUTH COLLEGE
HANOVER, NH 03755

KLOSKY, JAMES , PHD
ASSOCIATE MEMBER
DEPARTMENT OF PSYCHOLOGY
ST JUDE CHILDREN'S RESEARCH HOSPITAL
MEMPHIS, TN 38105

LAUDENSLAGER, MARK L., PHD
PROFESSOR
DIRECTOR, BEHAVIORAL IMMUNOLOGY
AND ENDOCRINOLOGY LABORATORY
DEPARTMENT OF PSYCHIATRY
UNIVERSITY OF COLORADO DENVER
AURORA, CO 80045

LAWSON, MICHAEL J, MD
SENIOR GASTROENTEROLOGIST
DEPARTMENT OF GASTROENTEROLOGY
SACRAMENTO MEDICAL CENTER
KAISER PERMANENTE
SACREMENTO, CA 95825

LAZOVICH, DEANN , PHD
ASSOCIATE PROFESSOR
DIVISION OF EPIDEMIOLOGY AND COMMUNITY HEALTH
SCHOOL OF PUBLIC HEALTH
UNIVERSITY OF MINNESOTA
MINNEAPOLIS, MN 554541015

LERMAN, CARYN , PHD
MARY W. CALKINS PROFESSOR
DIRECTOR, TOBACCO USE RESEARCH CENTER
DEPARTMENT OF PSYCHIATRY
UNIVERSITY OF PENNSYLVANIA
PHILADELPHIA, PA 19104

LOCHER, JULIE L., PHD
PROFESSOR
DEPARTMENT OF MEDICINE
UNIVERSITY OF ALABAMA, BIRMINGHAM
BIRMINGHAM, AL 35294

MULLAN, PATRICIA BRIDGET, PHD
PROFESSOR
DEPARTMENT OF MEDICAL EDUCATION
SCHOOL OF MEDICINE
UNIVERSITY OF MICHIGAN
ANN ARBOR, MI 48109

PADILLA, GERALDINE V, PHD
PROFESSOR EMERITUS
SCHOOL OF NURSING
UNIVERSITY OF CALIFORNIA, SAN FRANCISCO
SAN FRANCISCO, CA 94143

RABER, JACOB , PHD
PROFESSOR
DEPARTMENTS OF BEHAVIORAL
NEUROSCIENCE AND NEUROLOGY
OREGON HEALTH AND SCIENCE UNIVERSITY
PORTLAND, OR 97239

READER, STEVEN , PHD
ASSOCIATE PROFESSOR
DEPARTMENT OF GEOGRAPHY ENVIRONMENT &
PLANNING
SCHOOL OF GEOSCIENCES
UNIVERSITY OF SOUTH FLORIDA
TAMPA, FL 33620

SCHOEN, ROBERT E., MD, PHD
PROFESSOR
DIVISION OF GASTROENTEROLOGY, HEPATOLOGY
AND NUTRITION
UPMC PRESBYTERIAN
UNIVERSITY OF PITTSBURGH
PITTSBURGH, PA 15213

WISNIVESKY, JUAN P, MD, DPH
PROFESSOR
DEPARTMENT OF MEDICINE
MOUNT SINAI SCHOOL OF MEDICINE
NEW YORK, NY 10029

ZHENG, TONGZHANG , SCD, MD
PROFESSOR
DEPARTMENT OF ENVIRONMENTAL HEALTH SCIENCES
YALE UNIVERSITY, SCHOOL OF PUBLIC HEALTH
NEW HAVEN, CT 06520

MAIL REVIEWER(S)

POSTOLACHE, TEODOR T, MD
PROFESSOR AND DIRECTOR
MOOD AND ANXIETY PROGRAM
DEPARTMENT OF PSYCHIATRY
UNIVERSITY OF MARYLAND
BALTIMORE, MD 21201

SCIENTIFIC REVIEW ADMINISTRATOR

STOICA, ADRIANA , PHD
SCIENTIFIC REVIEW OFFICER
RESOURCES AND TRAINING REVIEW BRANCH
DIVISION OF EXTRAMURAL ACTIVITIES
NATIONAL CANCER INSTITUTE
NATIONAL INSTITUTES OF HEALTH
BETHESDA, MD 208929750

GRANTS TECHNICAL ASSISTANT

HESTER, SHEILA
PROGRAM SPECIALIST
RESOURCES AND TRAINING REVIEW BRANCH
DIVISION OF EXTRAMURAL ACTIVITIES
NATIONAL CANCER INSTITUTE
NATIONAL INSTITUTES OF HEALTH
BETHESDA, MD 208929750

Consultants are required to absent themselves from the room during the review of any application if their presence would constitute or appear to constitute a conflict of interest.
